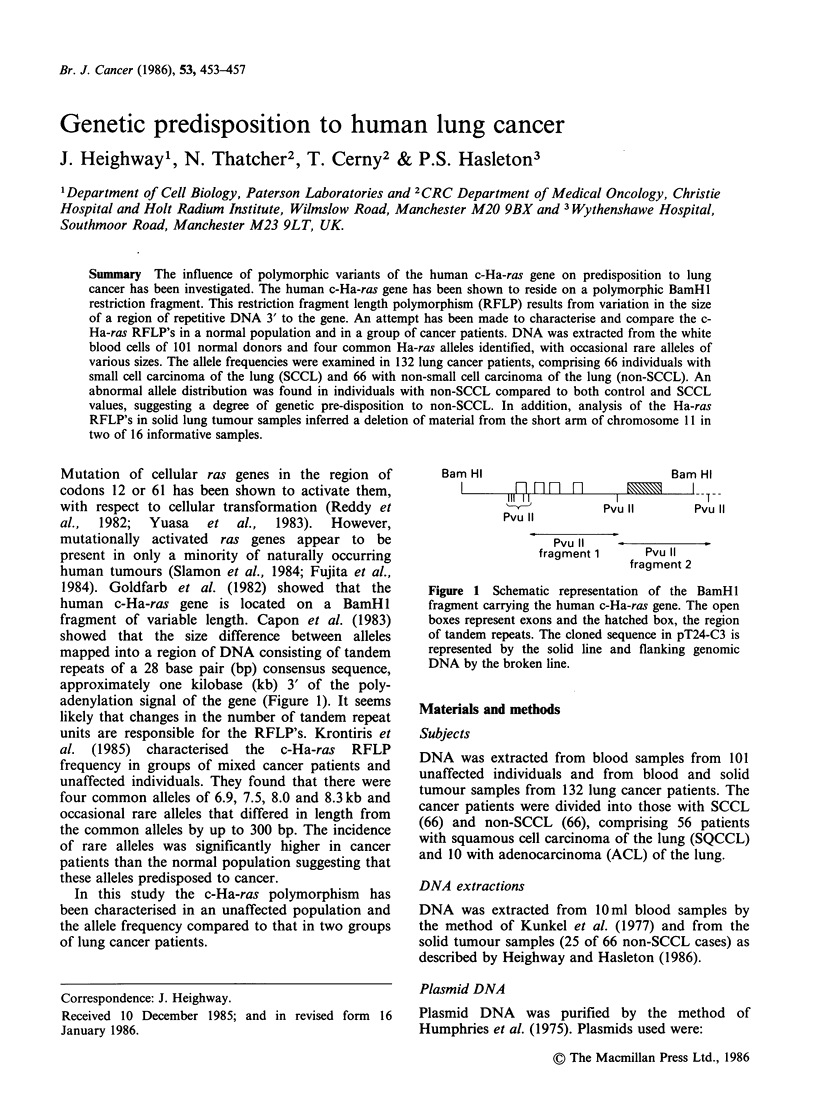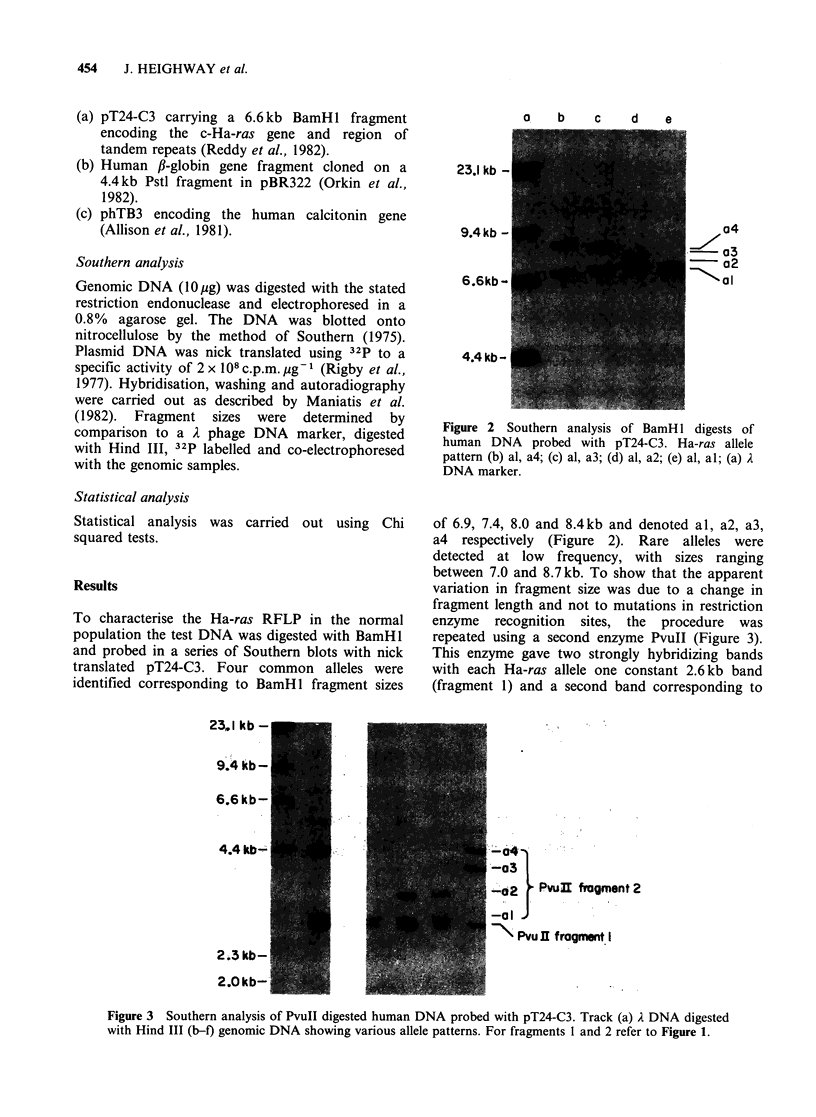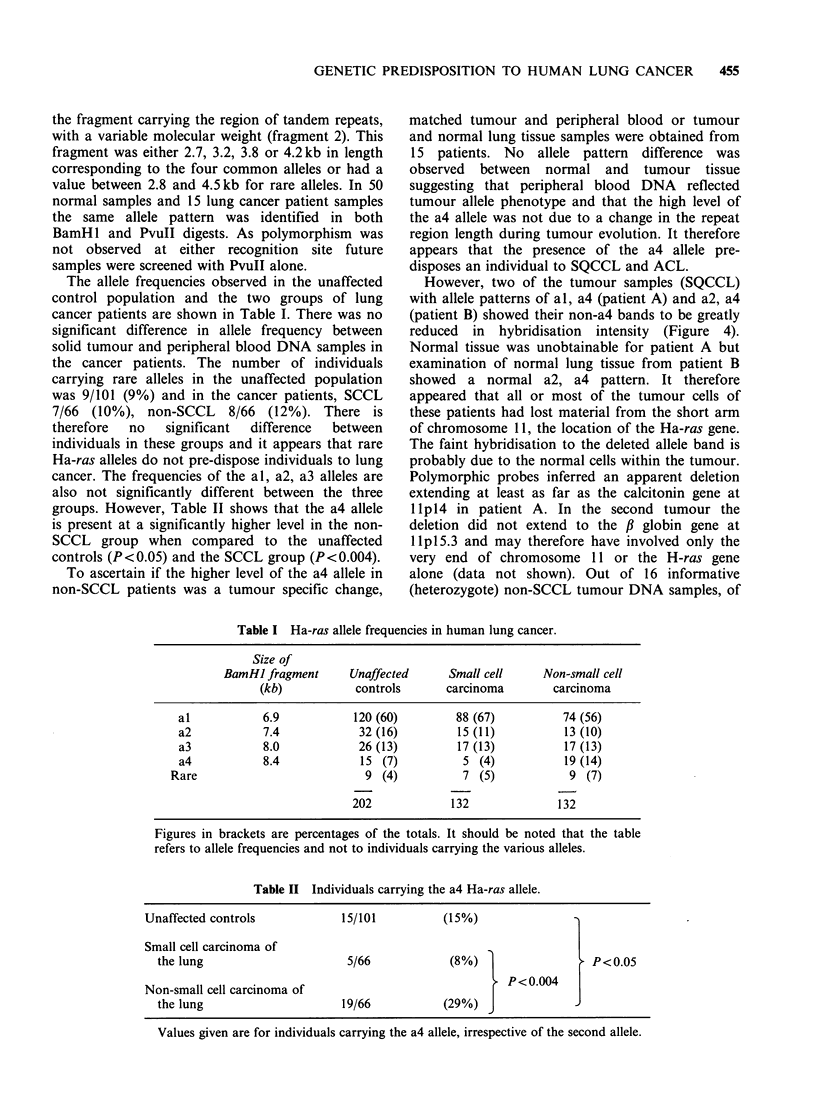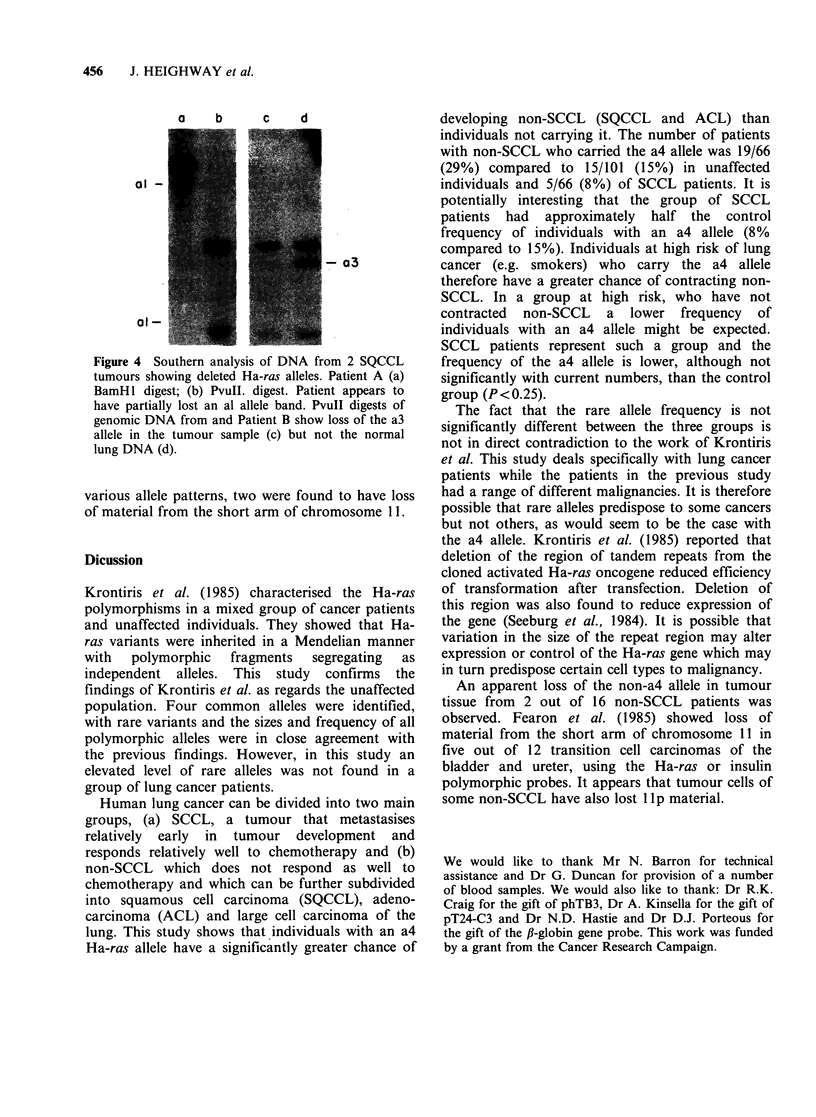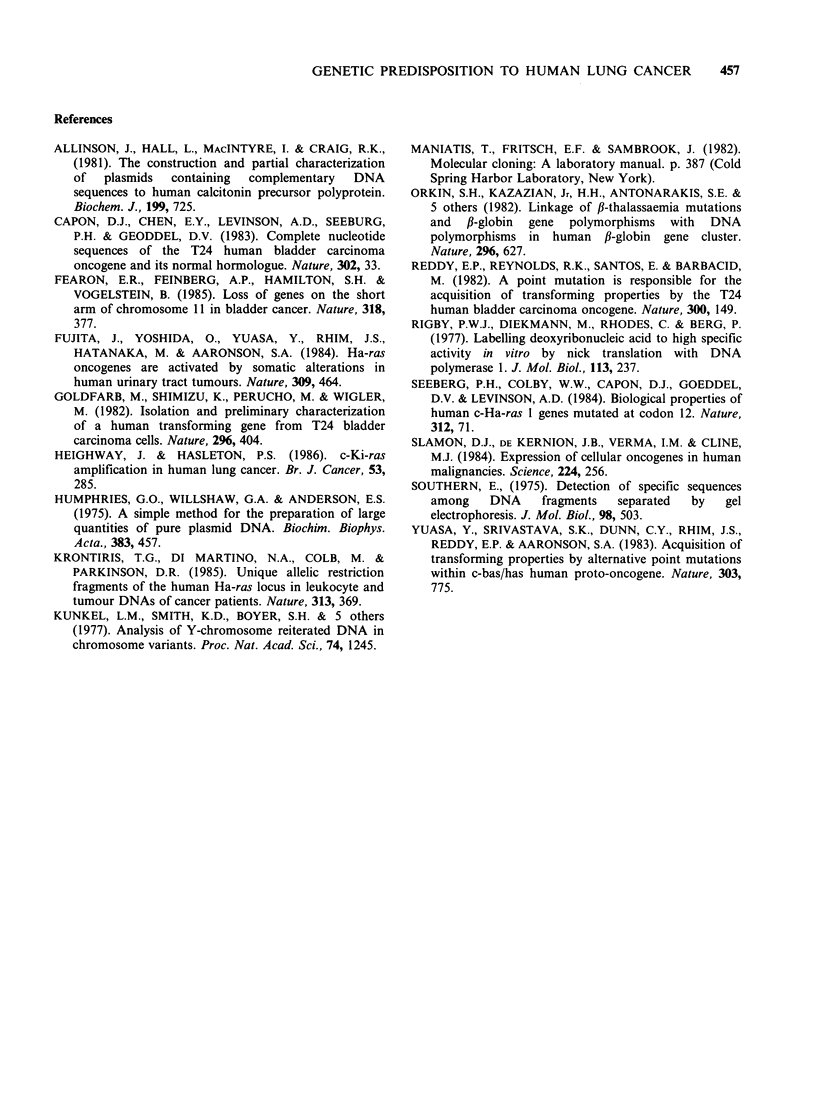# Genetic predisposition to human lung cancer.

**DOI:** 10.1038/bjc.1986.72

**Published:** 1986-04

**Authors:** J. Heighway, N. Thatcher, T. Cerny, P. S. Hasleton

## Abstract

**Images:**


*Br. J. Cancer* (1986), **53**, 453–457

### Genetic predisposition to human lung cancer

J. Heighway[1], N. Thatcher[2], T. Cerny[2] & P.S. Hasleton[3]

[1]*Department of Cell Biology, Paterson Laboratories and* [2]*CRC Department of Medical Oncology, Christie Hospital and Holt Radium Institute, Wilmslow Road, Manchester M20 9BX and* [3]*Wythenshawe Hospital, Southmoor Road, Manchester M23 9LT, UK.*

**Summary** The influence of polymorphic variants of the human c-Ha-*ras* gene on predisposition to lung cancer has been investigated. The human c-Ha-*ras* gene has been shown to reside on a polymorphic BamH1 restriction fragment. This restriction fragment length polymorphism (RFLP) results from variation in the size of a region of repetitive DNA 3′ to the gene. An attempt has been made to characterise and compare the c-Ha-*ras* RFLP's in a normal population and in a group of cancer patients. DNA was extracted from the white blood cells of 101 normal donors and four common Ha-*ras* alleles identified, with occasional rare alleles of various sizes. The allele frequencies were examined in 132 lung cancer patients, comprising 66 individuals with small cell carcinoma of the lung (SCCL) and 66 with non-small cell carcinoma of the lung (non-SCCL). An abnormal allele distribution was found in individuals with non-SCCL compared to both control and SCCL values, suggesting a degree of genetic pre-disposition to non-SCCL. In addition, analysis of the Ha-*ras* RFLP's in solid lung tumour samples inferred a deletion of material from the short arm of chromosome 11 in two of 16 informative samples.

Mutation of cellular *ras* genes in the region of codons 12 or 61 has been shown to activate them, with respect to cellular transformation (Reddy *et al.*, 1982; Yuasa *et al.*, 1983). However, mutationally activated *ras* genes appear to be present in only a minority of naturally occurring human tumours (Slamon *et al.*, 1984; Fujita *et al.*, 1984). Goldfarb *et al.* (1982) showed that the human c-Ha-*ras* gene is located on a BamH1 fragment of variable length. Capon *et al.* (1983) showed that the size difference between alleles mapped into a region of DNA consisting of tandem repeats of a 28 base pair (bp) consensus sequence, approximately one kilobase (kb) 3′ of the poly-adenylation signal of the gene (Figure 1). It seems likely that changes in the number of tandem repeat units are responsible for the RFLP's. Krontiris *et al.* (1985) characterised the c-Ha-*ras* RFLP frequency in groups of mixed cancer patients and unaffected individuals. They found that there were four common alleles of 6.9, 7.5, 8.0 and 8.3 kb and occasional rare alleles that differed in length from the common alleles by up to 300 bp. The incidence of rare alleles was significantly higher in cancer patients than the normal population suggesting that these alleles predisposed to cancer.

In this study the c-Ha-*ras* polymorphism has been characterised in an unaffected population and the allele frequency compared to that in two groups of lung cancer patients.

Correspondence: J. Heighway.

Received 10 December 1985; and in revised form 16 January 1986.

**Figure 1** Schematic representation of the BamH1 fragment carrying the human c-Ha-*ras* gene. The open boxes represent exons and the hatched box, the region of tandem repeats. The cloned sequence in pT24-C3 is represented by the solid line and flanking genomic DNA by the broken line.

### Materials and methods

### *Subjects*

DNA was extracted from blood samples from 101 unaffected individuals and from blood and solid tumour samples from 132 lung cancer patients. The cancer patients were divided into those with SCCL (66) and non-SCCL (66), comprising 56 patients with squamous cell carcinoma of the lung (SQCCL) and 10 with adenocarcinoma (ACL) of the lung.

### *DNA extractions*

DNA was extracted from 10 ml blood samples by the method of Kunkel *et al.* (1977) and from the solid tumour samples (25 of 66 non-SCCL cases) as described by Heighway and Hasleton (1986).

### *Plasmid DNA*

Plasmid DNA was purified by the method of Humphries *et al.* (1975). Plasmids used were:

© The Macmillan Press Ltd., 1986

(a) pT24-C3 carrying a 6.6 kb BamH1 fragment encoding the c-Ha-*ras* gene and region of tandem repeats (Reddy *et al.*, 1982).
(b) Human $\beta$-globin gene fragment cloned on a 4.4 kb Pstl fragment in pBR322 (Orkin *et al.*, 1982).
(c) phTB3 encoding the human calcitonin gene (Allison *et al.*, 1981).

*Southern analysis*

Genomic DNA (10 $\mu$g) was digested with the stated restriction endonuclease and electrophoresed in a 0.8% agarose gel. The DNA was blotted onto nitrocellulose by the method of Southern (1975). Plasmid DNA was nick translated using $^{32}$P to a specific activity of $2 \times 10^8$ c.p.m. $\mu g^{-1}$ (Rigby *et al.*, 1977). Hybridisation, washing and autoradiography were carried out as described by Maniatis *et al.* (1982). Fragment sizes were determined by comparison to a $\lambda$ phage DNA marker, digested with Hind III, $^{32}$P labelled and co-electrophoresed with the genomic samples.

**Figure 2** Southern analysis of BamH1 digests of human DNA probed with pT24-C3. Ha-*ras* allele pattern (b) al, a4; (c) al, a3; (d) al, a2; (e) al, al; (a) $\lambda$ DNA marker.

*Statistical analysis*

Statistical analysis was carried out using Chi squared tests.

### Results

To characterise the Ha-*ras* RFLP in the normal population the test DNA was digested with BamH1 and probed in a series of Southern blots with nick translated pT24-C3. Four common alleles were identified corresponding to BamH1 fragment sizes of 6.9, 7.4, 8.0 and 8.4 kb and denoted a1, a2, a3, a4 respectively (Figure 2). Rare alleles were detected at low frequency, with sizes ranging between 7.0 and 8.7 kb. To show that the apparent variation in fragment size was due to a change in fragment length and not to mutations in restriction enzyme recognition sites, the procedure was repeated using a second enzyme PvuII (Figure 3). This enzyme gave two strongly hybridizing bands with each Ha-*ras* allele one constant 2.6 kb band (fragment 1) and a second band corresponding to

**Figure 3** Southern analysis of PvuII digested human DNA probed with pT24-C3. Track (a) $\lambda$ DNA digested with Hind III (b–f) genomic DNA showing various allele patterns. For fragments 1 and 2 refer to **Figure 1**.

the fragment carrying the region of tandem repeats, with a variable molecular weight (fragment 2). This fragment was either 2.7, 3.2, 3.8 or 4.2 kb in length corresponding to the four common alleles or had a value between 2.8 and 4.5 kb for rare alleles. In 50 normal samples and 15 lung cancer patient samples the same allele pattern was identified in both BamH1 and PvuII digests. As polymorphism was not observed at either recognition site future samples were screened with PvuII alone.

The allele frequencies observed in the unaffected control population and the two groups of lung cancer patients are shown in Table I. There was no significant difference in allele frequency between solid tumour and peripheral blood DNA samples in the cancer patients. The number of individuals carrying rare alleles in the unaffected population was 9/101 (9%) and in the cancer patients, SCCL 7/66 (10%), non-SCCL 8/66 (12%). There is therefore no significant difference between individuals in these groups and it appears that rare Ha-*ras* alleles do not pre-dispose individuals to lung cancer. The frequencies of the a1, a2, a3 alleles are also not significantly different between the three groups. However, Table II shows that the a4 allele is present at a significantly higher level in the non-SCCL group when compared to the unaffected controls ($P<0.05$) and the SCCL group ($P<0.004$).

To ascertain if the higher level of the a4 allele in non-SCCL patients was a tumour specific change, matched tumour and peripheral blood or tumour and normal lung tissue samples were obtained from 15 patients. No allele pattern difference was observed between normal and tumour tissue suggesting that peripheral blood DNA reflected tumour allele phenotype and that the high level of the a4 allele was not due to a change in the repeat region length during tumour evolution. It therefore appears that the presence of the a4 allele predisposes an individual to SQCCL and ACL.

However, two of the tumour samples (SQCCL) with allele patterns of a1, a4 (patient A) and a2, a4 (patient B) showed their non-a4 bands to be greatly reduced in hybridisation intensity (Figure 4). Normal tissue was unobtainable for patient A but examination of normal lung tissue from patient B showed a normal a2, a4 pattern. It therefore appeared that all or most of the tumour cells of these patients had lost material from the short arm of chromosome 11, the location of the Ha-*ras* gene. The faint hybridisation to the deleted allele band is probably due to the normal cells within the tumour. Polymorphic probes inferred an apparent deletion extending at least as far as the calcitonin gene at 11p14 in patient A. In the second tumour the deletion did not extend to the $\beta$ globin gene at 11p15.3 and may therefore have involved only the very end of chromosome 11 or the H-*ras* gene alone (data not shown). Out of 16 informative (heterozygote) non-SCCL tumour DNA samples, of

**Table I** Ha-*ras* allele frequencies in human lung cancer.

| | *Size of BamH1 fragment (kb)* | *Unaffected* controls | *Small cell* carcinoma | *Non-small cell* carcinoma |
|---|---|---|---|---|
| a1 | 6.9 | 120 (60) | 88 (67) | 74 (56) |
| a2 | 7.4 | 32 (16) | 15 (11) | 13 (10) |
| a3 | 8.0 | 26 (13) | 17 (13) | 17 (13) |
| a4 | 8.4 | 15 (7) | 5 (4) | 19 (14) |
| Rare | | 9 (4) | 7 (5) | 9 (7) |
| | | 202 | 132 | 132 |

Figures in brackets are percentages of the totals. It should be noted that the table refers to allele frequencies and not to individuals carrying the various alleles.

**Table II** Individuals carrying the a4 Ha-*ras* allele.

| | | | | |
|---|---|---|---|---|
| Unaffected controls | 15/101 | (15%) | | $P<0.05$ (vs. non-small cell) |
| Small cell carcinoma of the lung | 5/66 | (8%) | $P<0.004$ (vs. non-small cell) | |
| Non-small cell carcinoma of the lung | 19/66 | (29%) | | |

Values given are for individuals carrying the a4 allele, irrespective of the second allele.

**Figure 4** Southern analysis of DNA from 2 SQCCL tumours showing deleted Ha-*ras* alleles. Patient A (a) BamH1 digest; (b) PvuII. digest. Patient appears to have partially lost an al allele band. PvuII digests of genomic DNA from and Patient B show loss of the a3 allele in the tumour sample (c) but not the normal lung DNA (d).

various allele patterns, two were found to have loss of material from the short arm of chromosome 11.

### Dicussion

Krontiris *et al.* (1985) characterised the Ha-*ras* polymorphisms in a mixed group of cancer patients and unaffected individuals. They showed that Ha-*ras* variants were inherited in a Mendelian manner with polymorphic fragments segregating as independent alleles. This study confirms the findings of Krontiris *et al.* as regards the unaffected population. Four common alleles were identified, with rare variants and the sizes and frequency of all polymorphic alleles were in close agreement with the previous findings. However, in this study an elevated level of rare alleles was not found in a group of lung cancer patients.

Human lung cancer can be divided into two main groups, (a) SCCL, a tumour that metastasises relatively early in tumour development and responds relatively well to chemotherapy and (b) non-SCCL which does not respond as well to chemotherapy and which can be further subdivided into squamous cell carcinoma (SQCCL), adeno-carcinoma (ACL) and large cell carcinoma of the lung. This study shows that individuals with an a4 Ha-*ras* allele have a significantly greater chance of developing non-SCCL (SQCCL and ACL) than individuals not carrying it. The number of patients with non-SCCL who carried the a4 allele was 19/66 (29%) compared to 15/101 (15%) in unaffected individuals and 5/66 (8%) of SCCL patients. It is potentially interesting that the group of SCCL patients had approximately half the control frequency of individuals with an a4 allele (8% compared to 15%). Individuals at high risk of lung cancer (e.g. smokers) who carry the a4 allele therefore have a greater chance of contracting non-SCCL. In a group at high risk, who have not contracted non-SCCL a lower frequency of individuals with an a4 allele might be expected. SCCL patients represent such a group and the frequency of the a4 allele is lower, although not significantly with current numbers, than the control group ($P < 0.25$).

The fact that the rare allele frequency is not significantly different between the three groups is not in direct contradiction to the work of Krontiris *et al.* This study deals specifically with lung cancer patients while the patients in the previous study had a range of different malignancies. It is therefore possible that rare alleles predispose to some cancers but not others, as would seem to be the case with the a4 allele. Krontiris *et al.* (1985) reported that deletion of the region of tandem repeats from the cloned activated Ha-*ras* oncogene reduced efficiency of transformation after transfection. Deletion of this region was also found to reduce expression of the gene (Seeburg *et al.*, 1984). It is possible that variation in the size of the repeat region may alter expression or control of the Ha-*ras* gene which may in turn predispose certain cell types to malignancy.

An apparent loss of the non-a4 allele in tumour tissue from 2 out of 16 non-SCCL patients was observed. Fearon *et al.* (1985) showed loss of material from the short arm of chromosome 11 in five out of 12 transition cell carcinomas of the bladder and ureter, using the Ha-*ras* or insulin polymorphic probes. It appears that tumour cells of some non-SCCL have also lost 11p material.

We would like to thank Mr N. Barron for technical assistance and Dr G. Duncan for provision of a number of blood samples. We would also like to thank: Dr R.K. Craig for the gift of phTB3, Dr A. Kinsella for the gift of pT24-C3 and Dr N.D. Hastie and Dr D.J. Porteous for the gift of the $\beta$-globin gene probe. This work was funded by a grant from the Cancer Research Campaign.

### References

ALLINSON, J., HALL, L., MACINTYRE, I. & CRAIG, R.K., (1981). The construction and partial characterization of plasmids containing complementary DNA sequences to human calcitonin precursor polyprotein. *Biochem. J.*, **199**, 725.

CAPON, D.J., CHEN, E.Y., LEVINSON, A.D., SEEBURG, P.H. & GEODDEL, D.V. (1983). Complete nucleotide sequences of the T24 human bladder carcinoma oncogene and its normal hormologue. *Nature*, **302**, 33.

FEARON, E.R., FEINBERG, A.P., HAMILTON, S.H. & VOGELSTEIN, B. (1985). Loss of genes on the short arm of chromosome 11 in bladder cancer. *Nature*, **318**, 377.

FUJITA, J., YOSHIDA, O., YUASA, Y., RHIM, J.S., HATANAKA, M. & AARONSON, S.A. (1984). Ha-*ras* oncogenes are activated by somatic alterations in human urinary tract tumours. *Nature*, **309**, 464.

GOLDFARB, M., SHIMIZU, K., PERUCHO, M. & WIGLER, M. (1982). Isolation and preliminary characterization of a human transforming gene from T24 bladder carcinoma cells. *Nature*, **296**, 404.

HEIGHWAY, J. & HASLETON, P.S. (1986). c-Ki-*ras* amplification in human lung cancer. *Br. J. Cancer*, **53**, 285.

HUMPHRIES, G.O., WILLSHAW, G.A. & ANDERSON, E.S. (1975). A simple method for the preparation of large quantities of pure plasmid DNA. *Biochim. Biophys. Acta.*, **383**, 457.

KRONTIRIS, T.G., DI MARTINO, N.A., COLB, M. & PARKINSON, D.R. (1985). Unique allelic restriction fragments of the human Ha-*ras* locus in leukocyte and tumour DNAs of cancer patients. *Nature*, **313**, 369.

KUNKEL, L.M., SMITH, K.D., BOYER, S.H. & 5 others (1977). Analysis of Y-chromosome reiterated DNA in chromosome variants. *Proc. Nat. Acad. Sci.*, **74**, 1245.

MANIATIS, T., FRITSCH, E.F. & SAMBROOK, J. (1982). Molecular cloning: A laboratory manual. p. 387 (Cold Spring Harbor Laboratory, New York).

ORKIN, S.H., KAZAZIAN, Jr, H.H., ANTONARAKIS, S.E. & 5 others (1982). Linkage of $\beta$-thalassaemia mutations and $\beta$-globin gene polymorphisms with DNA polymorphisms in human $\beta$-globin gene cluster. *Nature*, **296**, 627.

REDDY, E.P., REYNOLDS, R.K., SANTOS, E. & BARBACID, M. (1982). A point mutation is responsible for the acquisition of transforming properties by the T24 human bladder carcinoma oncogene. *Nature*, **300**, 149.

RIGBY, P.W.J., DIEKMANN, M., RHODES, C. & BERG, P. (1977). Labelling deoxyribonucleic acid to high specific activity *in vitro* by nick translation with DNA polymerase 1. *J. Mol. Biol.*, **113**, 237.

SEEBERG, P.H., COLBY, W.W., CAPON, D.J., GOEDDEL, D.V. & LEVINSON, A.D. (1984). Biological properties of human c-Ha-*ras* 1 genes mutated at codon 12. *Nature*, **312**, 71.

SLAMON, D.J., DE KERNION, J.B., VERMA, I.M. & CLINE, M.J. (1984). Expression of cellular oncogenes in human malignancies. *Science*, **224**, 256.

SOUTHERN, E., (1975). Detection of specific sequences among DNA fragments separated by gel electrophoresis. *J. Mol. Biol.*, **98**, 503.

YUASA, Y., SRIVASTAVA, S.K., DUNN, C.Y., RHIM, J.S., REDDY, E.P. & AARONSON, S.A. (1983). Acquisition of transforming properties by alternative point mutations within c-bas/has human proto-oncogene. *Nature*, **303**, 775.